# Domain-Independent Inhibition of CBP/p300 Attenuates α-Synuclein Aggregation

**DOI:** 10.1021/acschemneuro.1c00215

**Published:** 2021-06-10

**Authors:** Irena Hlushchuk, Heikki Ruskoaho, Andrii Domanskyi, Mikko Airavaara, Mika J. Välimäki

**Affiliations:** † Drug Research Program, Division of Pharmacology and Pharmacotherapy, Faculty of Pharmacy, University of Helsinki, Viikinkaari 5 E, Helsinki FI-00014, Finland; ‡ Institute of Biotechnology, HiLIFE, University of Helsinki, Viikinkaari 5 D, Helsinki FI-00014, Finland; § Neuroscience Center, HiLIFE, University of Helsinki, Haartmaninkatu 8, Helsinki FI-00290, Finland

**Keywords:** α-Synuclein, lysine acetyltransferase, bromodomain, Parkinson’s disease, CBP, p300

## Abstract

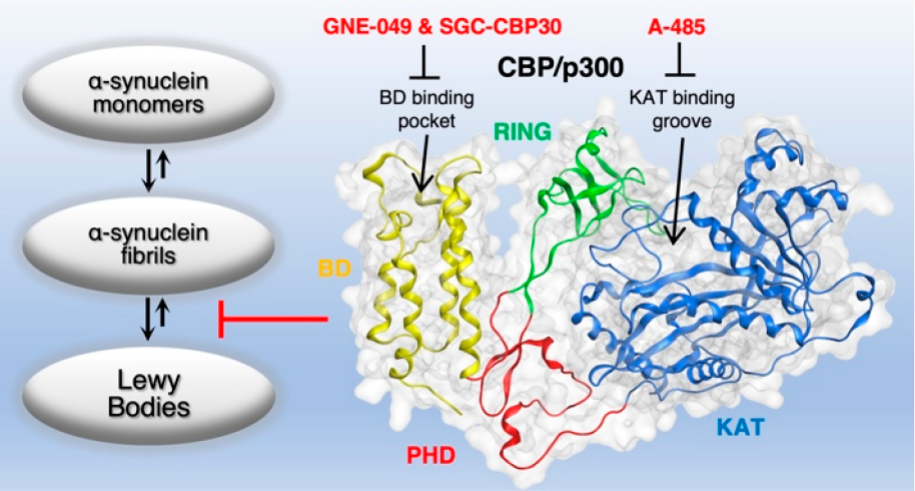

Neurodegenerative diseases are associated with failed proteostasis and accumulation of insoluble protein aggregates that compromise neuronal function and survival. In Parkinson’s disease, a major pathological finding is Lewy bodies and neurites that are mainly composed of phosphorylated and aggregated α-synuclein and fragments of organelle membranes. Here, we analyzed a series of selective inhibitors acting on multidomain proteins CBP and p300 that contain both lysine acetyltransferase and bromodomains and are responsible for the recognition and enzymatic modification of lysine residues. By using high-affinity inhibitors, A-485, GNE-049, and SGC-CBP30, we explored the role of two closely related proteins, CBP and p300, as promising targets for selective attenuation of α-synuclein aggregation. Our data show that selective CBP/p300 inhibitors may alter the course of pathological α-synuclein accumulation in primary mouse embryonic dopaminergic neurons. Hence, drug-like CBP/p300 inhibitors provide an effective approach for the development of high-affinity drug candidates preventing α-synuclein aggregation via systemic administration.

Intracellular Lewy bodies and neurites are common pathophysiological hallmarks in Parkinson’s disease (PD) patient brains as well as in dementia with Lewy bodies. Their major component is aggregated α-synuclein. Lewy body and neurite pathology affects many regions of the nervous system and various neuronal phenotypes.^1−3^ The degeneration and progressive loss of dopaminergic neurons in the substantia nigra pars compacta is associated with PD’s classic major motor symptoms.^3^ The predominant component in the formation of Lewy bodies, pale bodies, and Lewy neurites^4^ is α-synuclein, abundantly present in human brains but able to promote cellular toxicity when aggregated.^5^ Lewy bodies are α-synuclein immunoreactive neuronal inclusions, consisting of membranous and vesicular structures, malformed organelles, and neurofilaments.^6^

α-Synuclein is a lysine-rich protein (15 out of 140 amino acids, lysine content of 10.7%) and is localized predominantly within the cytosol of neurons as an intrinsically disordered monomer.^7^ Abnormal α-synuclein folding, aggregation, and accumulation of various oligomeric polyforms has been associated with the pathogenesis of PD. Previous studies demonstrate that α-synuclein gene (SNCA) locus duplication or triplication and single-point mutations including A30P, E46K, H50Q, G51D, A53T, A53E, and A53V are associated with α-synuclein dysfunction and aggregation and lead to either familial or early onset of PD.^8−10^ Various heterogeneous α-synuclein structural polymorphs have been identified by cryo-EM in artificial conditions, indicating a large variability of α-synuclein fibril topologies in cells. Prominent wild-type α-synuclein structural kernels involve rod-type (type 1a, i.e., 6h6b)^11,12^ and twister-type (type 1b, i.e., 6cu8)^13^ polyforms that express zipper-like binding interfaces and monomeric geometry with rotational symmetry along the fibril axis. The hydrophobic region of α-synuclein protein (residues 50–57) folds into a β-strand and forms an interface important for dimerization and fibril formation.^12^ Accordingly, disease-relevant hereditary α-synuclein mutations present at the dimer interface of the fibril (H50Q, G51D, and A53T/E) or involved in the stabilization of the protofilament (E46K) stand out as unique structural conformations distinct from those of the wild-type protein.^12−15^

Previous studies have confirmed that the folding and stability of wild-type α-synuclein oligomers may depend on cellular and experimental conditions such as post-translational modifications (PTMs), chemical substances, and buffer ion compositions.^16^ α-Synuclein fibrils are prone to PTMs (phosphorylation, acetylation, ubiquitination) and conformational rearrangements associated with the maturation of pathological Lewy body inclusions that trigger the enhanced interactome with cytosolic proteins and cellular organelles.^17^ Considering the chemical substances, high-throughput screening campaigns identified inhibitors of α-synuclein aggregation such as polyanionic small molecule SynuClean-D and its derivative,^18^ anle138b,^19^ and NPT200-11,^20^ and the latter two are being tested in clinical trials.^21,22^ Alterations in buffer selection and associated counterions influence α-synuclein conformational promiscuity, further endorsing the impact of small changes in total energy among the different α-synuclein polyforms.^23^ Cryo-EM of wild-type polyform 1a illustrates the clusters of three lysine residues in close proximity under phosphate buffer (Figure 1A). In those conditions, Guerrero-Ferreira and colleagues^11^ observed an enhanced density in the cryo-EM map, indicating a polyanionic phosphate that may neutralize the repulsion of three positively charged residues, thus clarifying the molecular mechanism and energy barrier to overcome prior the fibril formation.^23^ This was further confirmed as completely unique α-synuclein polyforms 2a and 2b were obtained with phosphate-free buffers.

**Figure 1:**
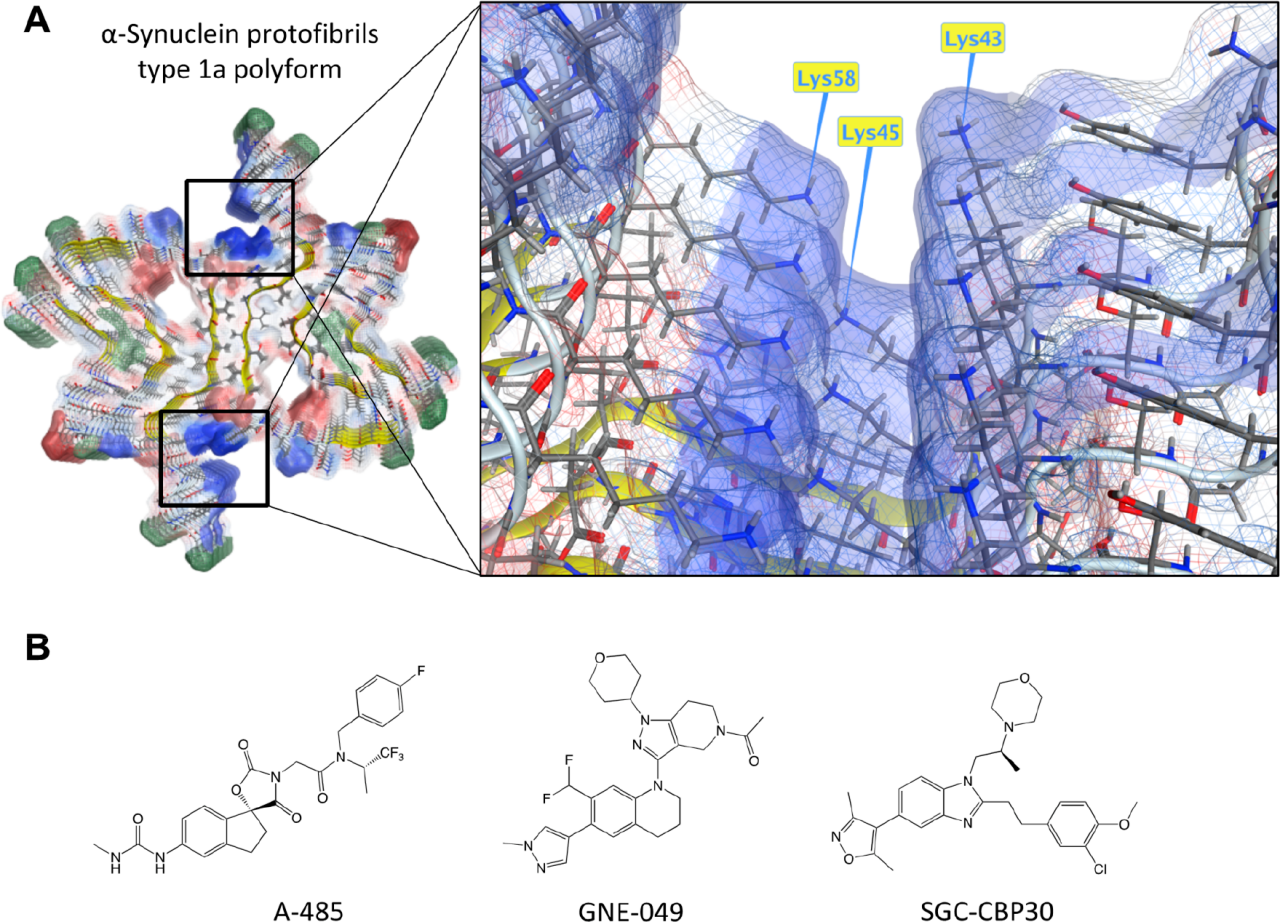
Apparent lysine clustering in α-synuclein protofilaments. (A) Type 1a polyform of α-synuclein fibril reveals a cluster of positively charged lysine residues at both ends of the α-synuclein binding interface, the same area where many of the disease-relevant mutations are expressed. Wild-type α-synuclein with unmodified lysine residues at positions Lys43, Lys45, and Lys58 presents a high internal repulsion contributing to protofibril formation. An electrostatic map indicates blue color for local over-representation of protons, red color for local under-representation of protons, and green color for hydrophobic batches. Template downloaded from PDB: 6h6b.^11^ (B) Lysine acetyltransferase (A-485) and bromodomain inhibitors (GNE-049, SGC-CBP30) used in this study.

Understanding how the seeding of α-synuclein aggregation with preformed fibrils is regulated under transformative conditions may prove valuable concerning the inclusive linkage to disease pathogenicity. Though the neuronal accumulation of misfolded α-synuclein in PD is apparent, the effect of lysine residues on molecular dynamics of α-synuclein misfolding and disease progression remains unclear. Pharmacological intervention offers a practical tool to explore the molecular mechanisms affecting α-synuclein aggregation. To this end, we systematically investigated the impact of lysine acetyltransferase (KAT) and bromodomain inhibitors on preformed fibril-induced α-synuclein aggregation in primary dopaminergic neurons. Interrogating KAT/bromodomain functions using high-affinity inhibitors offers an efficient target validation approach that potentially streamlines the pathway to clinical development.

In this study, we focused on the inhibitors of specific multidomain proteins carrying both KATs and bromodomains responsible for the recognition and enzymatic modification of lysine residues. Tested compounds include series of inhibitors for CBP/p300 (A-485, GNE-049, and SGC-CBP30, Figure 1B), SMARCA2/4 (PFI-3), TATA-box binding associated protein 1 (TAF1) (3i-5001), and bromodomains and extra-terminal domain (BET) family proteins ((+)-JQ1).

We have previously demonstrated that treatment with glial-cell-line-derived neurotrophic factor (GDNF) efficiently and robustly reduced phosphorylated α-synuclein accumulation in dopaminergic neurons.^24,25^ Primary cell cultures are considered optimal models to scrutinize neuronal pathology *in vitro*. In the midbrain culture of E-13 mouse, the number of dopaminergic neurons is high, about 5 to 10%, and about half of them survive until day *in vitro* (DIV)-5 in cell media without neurotrophic factors.^26^ The absence of neurotrophic factors is important for our experiment, since GDNF rescues dopaminergic neurons from α-synuclein aggregation. For that reason, we exploited GDNF as a positive control in the current experiment (Figure 2).^24,25^

**Figure 2:**
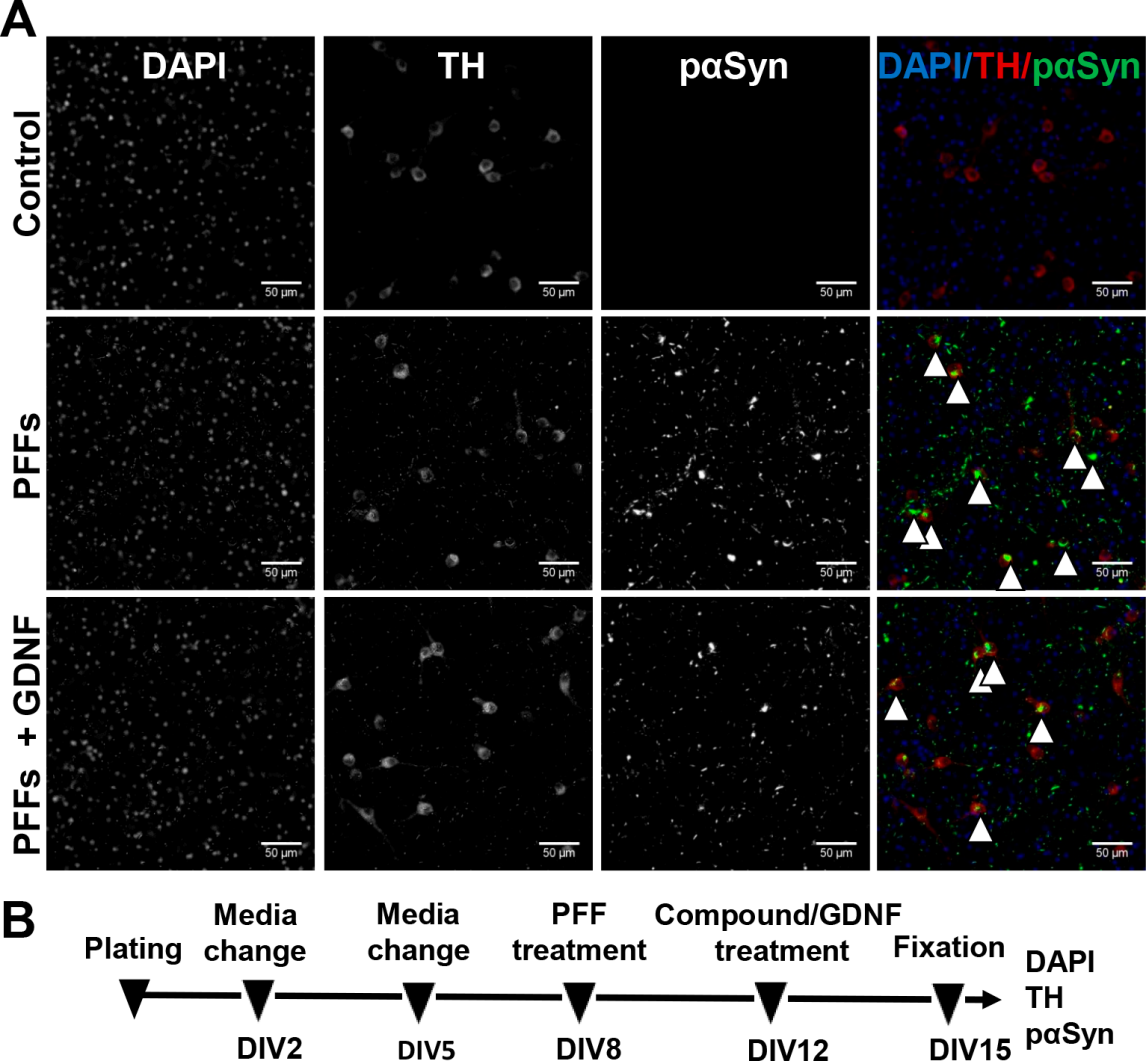
Accumulation of phosphorylated α-synuclein at Ser129 (pαSyn) in mouse embryonic dopaminergic neurons. (A) Treatment with preformed fibrils (PFFs) for 7 days induced formation of large intrasomal Lewy-body-like pαSyn-positive aggregates (indicated by arrowheads) in tyrosine hydroxylase (TH)-positive dopaminergic neurons, as assessed by immunofluorescent staining with corresponding antibodies. GDNF, added 4 days after PFFs, reduced the pαSyn aggregation and was used as a positive control in the experiments. (B) Treatment timeline. Scale bar, 50 μm.

Compound GNE-049 is a potent bromodomain inhibitor of CBP/p300, with nearly 4000-fold selectivity over bromodomain-containing protein 4 (BRD4). GNE-049 was added at DIV-12 to primary midbrain cultures pretreated with preformed fibrils (PFFs) at DIV-8. GNE-049 at all tested concentrations (0.1, 1, and 10 μM) decreased α-synuclein aggregation, similarly to positive control GDNF (*F* (4, 8) = 58.34; *P* < 0.0001, treatment effect and *F* (4, 8) = 56.96, *P* < 0.0001 for PFF × treatment interaction, two-way ANOVA followed by Holm-Sidak’s multiple-comparisons test: for GNE-049, 0.1 μM vs vehicle, *p* < 0.0001, *t* = 13.48, DF = 8; for GNE-049, 1 μM vs vehicle, *p* < 0.0001, *t* = 12.78, DF = 8; for GNE-049, 10 μM vs vehicle, *p* < 0.0001, *t* = 17.01, DF = 8; for GDNF vs vehicle, *p* < 0.0001, *t* = 19.58, DF = 8) (Figure 3A,C). Dopaminergic neuron survival evaluated by the number of tyrosine hydroxylase (TH)-positive cells was not affected either by GNE-049 or by PFF treatment (Figure 3B,C).

**Figure 3:**
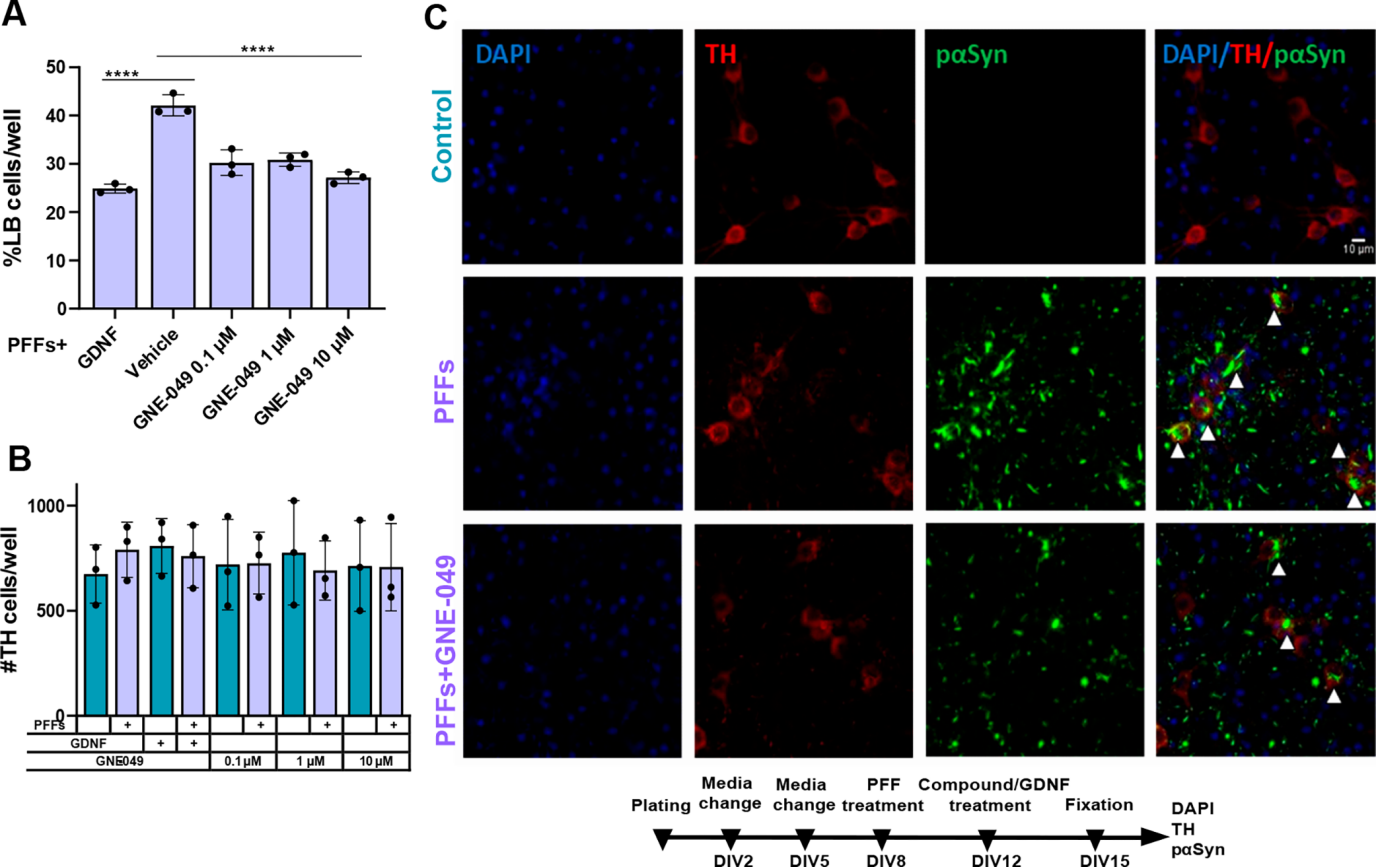
GNE-049 shows a significant effect on the phosphorylated α-synuclein aggregation in dopaminergic neurons. (A) GNE-049 at concentrations of 0.1, 1, and 10 μM added at DIV-12 to the PFF-pretreated primary dopaminergic cell culture significantly decreases formation of Lewy-body-like aggregates. Two-way repeated-measures (RM) ANOVA, Holm-Sidak’s multiple-comparison test, ****p < 0.0001, *n* = 3 independent experiments, each containing four wells per tested condition, as technical replicates. (B) Dopaminergic cell survival evaluated by the number of TH-positive cells was not affected by PFFs or by GNE-049 at concentrations of 0.1, 1, and 10 μM. (C) Representative images of control- (top), PFF- (middle), and PFF-and-GNE-049-treated (bottom) groups. Arrowheads point at α-synuclein accumulations in TH-positive neurons. Data are mean ± SD. Scale bar, 10 μm.

To further confirm the initial finding with the bromodomain inhibitor of CBP/p300, other compounds acting through the same molecular mechanism were evaluated. Already at 1 μM, compound SGC-CBP30 decreased PFF-induced α-synuclein aggregation in primary dopaminergic neurons (two-way ANOVA followed by Holm-Sidak’s multiple-comparisons test: for SGC-CBP30, 1 μM vs vehicle interaction, *p* = 0.0245, *t* = 3.997, DF = 12); at 10 μM, its effect was similar to positive control GDNF (*F* (4, 12) = 16.68; *P* < 0.0001, treatment effect and *F* (4, 12) = 16.66, *P* < 0.0001 for PFF × treatment interaction, two-way ANOVA followed by Holm-Sidak’s multiple-comparisons test: for SGC-CBP30, 10 μM vs vehicle, *p* < 0.0001, *t* = 8.904, DF = 12; for GDNF vs vehicle *p* < 0.0001, *t* = 8.89, DF = 12) (Figure 4A). SGC-CBP30 at concentrations of 0.1 and 1 μM showed no toxicity to TH-positive cells; however, at 10 μM, SGC-CBP30 caused loss of TH-positive cells but, curiously, not when combined with PFF treatment (*F* (4, 12) = 19.72; *P* < 0.0001, treatment effect, two-way ANOVA followed by Holm-Sidak’s multiple-comparisons test: for SGC-CBP30, 10 μM + PFFs vs vehicle, *p* = 0.2851, *t* = 3.032, DF = 12; for SGC-CBP30, 10 μM vs vehicle, *p* < 0.001, *t* = 6.586, DF = 12) (Figure 4B).

**Figure 4:**
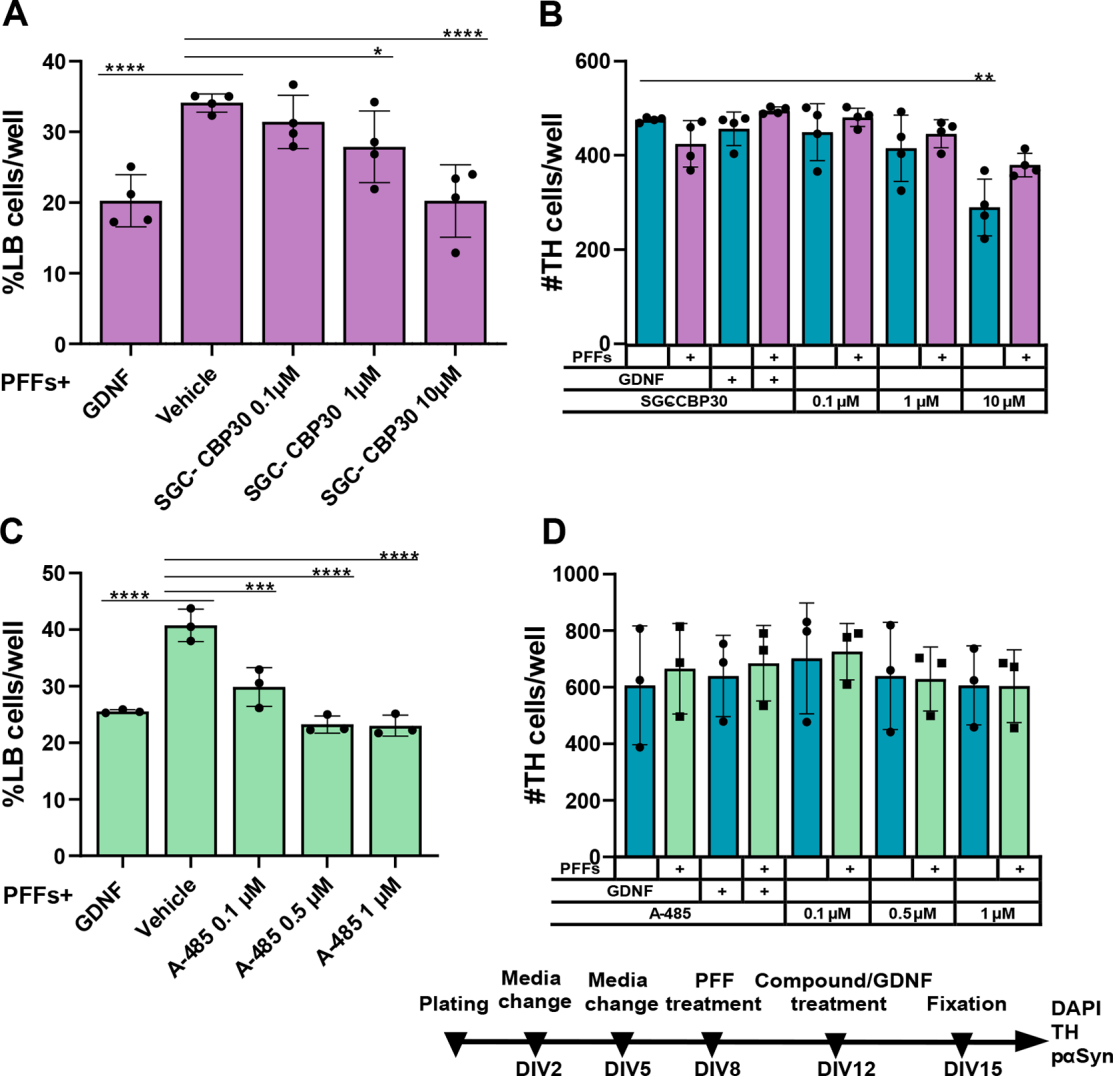
CBP/p300 inhibitor SGC-CBP30 at concentrations of 1 and 10 μM and A-485 at concentrations of 0.1, 0.5, and 1 μM significantly decrease the level of intracellular phosphorylated α-synuclein accumulation in dopaminergic neurons. (A) The effect of SGC-CBP30 on the formation of aggregates in dopaminergic neurons’ soma (two-way RM ANOVA, Holm-Sidak’s multiple-comparison test, ****p < 0.0001, **p < 0.01, *p < 0.05, *n* = 4 independent experiments, data are mean ± SD). Each independent experiment contained four wells per experimental condition as technical replicates. (B) Average number of TH-positive cells per well (two-way RM ANOVA, Holm-Sidak’s multiple-comparison test, **p < 0.01, *n* = 4 independent experiments, data are mean ± SD). (C) A-485 at concentrations of 0.1, 0.5, and 1 μM demonstrates a significant decrease of the phosphorylated α-synuclein aggregation in dopaminergic neurons (two-way RM ANOVA, Holm-Sidak’s multiple-comparison test, ****p < 0.0001, ***p < 0.001, *n* = 3 independent experiments, data are mean ± SD). Each independent experiment contained five wells per experimental condition as technical replicates. (D) Concentrations of A-485 used were not significantly toxic to dopaminergic neurons.

Next, we examined the role of the KAT domain of CBP/p300 in α-synuclein aggregation. Compound A-485 at concentrations of 0.1, 0.5, and 1 μM decreased α-synuclein aggregation to a similar extent as positive control GDNF (*F* (4, 8) = 48.05; *P* < 0.0001, treatment effect and *F* (4, 8) = 45.17, *P* < 0.0001 for PFF × treatment interaction, two-way ANOVA followed by Holm-Sidak’s multiple-comparisons test: for A-485, 0.1 μM vs vehicle, *p* < 0.001, *t* = 9.927, DF = 8; for A-485, 0.5 μM vs vehicle, *p* < 0.0001, *t* = 15.97, DF = 8; for A-485, 1 μM vs vehicle, *p* < 0.0001, *t* = 16.18, DF = 8; for GDNF vs vehicle, *p* < 0.0001, *t* = 13.87, DF = 8) (Figure 4C). At 0.1, 0.5, and 1 μM, A-485 did not reduce the number of TH-positive neurons (Figure 4D); however, higher concentrations of A-485 (10 μM) showed toxicity to the primary dopaminergic cell cultures (Supplementary Figure S1). Other bromodomain inhibitors of SMARCA2/4 (PFI-3), TAF1 (3i-5001), and BET family proteins ((+)-JQ1) showed little or no effect on α-synuclein aggregation and the number of TH-positive cells, highlighting the selective effect of CBP/p300 inhibition in dopaminergic neurons (Supplementary Figure S2). Preliminary experiments combining the protective substances GDNF and SGC-CBP30 showed neither added benefits to α-synuclein aggregation nor increased cellular toxicity (Supplementary Figure S3). However, simultaneous exposure to both A-485 and GNE-049 at concentrations of 0.1 and 1 μM demonstrated significant toxicity to the primary dopaminergic cell cultures (Supplementary Figure S4).

KAT and bromodomain proteins, classically linked to epigenetic regulation and chromatin remodeling, are promising target classes for drug discovery and development. Drug candidates acting on the BET class of bromodomains have demonstrated clinical potential in oncology indications.^27^ Here, we explored the effect of KAT/bromodomain inhibitors in cultured primary dopaminergic neurons and demonstrated for the first time that selective inhibitors of nuclear proteins CBP/p300, e.g., A-485, GNE-049, SGC-CBP30, can modulate α-synuclein aggregation in the cytosol. Strikingly, our results demonstrate that domain-independent inhibition of either the KAT or bromodomain of CBP/p300 at 100 nM is enough to block α-synuclein aggregation in neurons. A pilot experiment also indicated that simultaneous use of GDNF and SGC-CBP30 does not induce a synergistic effect. In this assay, bromodomain inhibitors of CBP/p300 were extremely well-tolerated by TH-positive cells. Moreover, a previous study shows that bromodomain inhibitors of CBP/p300 prevented amyloid-like protein aggregation in human cells.^28^

Transcriptional coactivators CBP and p300 (also known as CREBBP or KAT3A and EP300 or KAT3B, respectively) are two closely related proteins that coordinate and integrate multiple signal-dependent events with the transcriptional apparatus, affecting cellular proliferation, differentiation, and apoptosis.^29^ Catalytic KATs and noncatalytic bromodomains of CBP/p300 facilitate the binding of high-affinity ligands (e.g., A-485, GNE-049, and SGC-CBP30).^30−32^ GNE-049 (MW 511, cLogP 3.6, topological polar surface area (TPSA) 68.4) is a highly potent and selective bromodomain inhibitor of CBP/p300 (IC_50_ of 1.1 and 2.3 nM for CBP and p300, respectively). Previous study has shown that compound GNE-049 (250 mg/kg) penetrates the blood–brain barrier (unbound brain to unbound plasma concentration ratio, *K*p_uu_ = 0.43) and induces adverse central nervous system (CNS)-related signs (e.g., marked hyperactivity and vocalization) in rats.^30^ SGC-CBP30 is another selective inhibitor for the bromodomain of CBP/p300 (IC_50_ of 21 and 32 nM for CBP and p300, respectively). Molecular parameters of SGC-CBP30 (MW 509, cLogP 5.1, and TPSA 65.6) suggest its suitability for systemic administration. However, the number of tautomeric and ionization states of compound SGC-CBP30 at neutral conditions may hinder optimal penetration to the CNS. On the other hand, A-485 is a potent and selective inhibitor of the catalytic KAT domain of CBP/p300, with IC_50_ values of 9.8 and 2.6 nM for CBP and p300, respectively. Molecular parameters of A-485 (MW 536, cLogP 3.8, and TPSA 108.1) suggest compromised penetration to CNS. However, the delivery and penetration of compound A-485 across the blood–brain barrier is not known.

Progression of Lewy body pathology is a characteristic feature in PD patient brains and has been suggested to be caused by the self-templating properties of aggregated α-synuclein. The development of therapies preventing pathological α-synuclein aggregation could benefit from repurposing of the above-mentioned lead compounds. High doses of CBP/p300 inhibitors have been used in cancer research involving serious adverse effects.^30^ In contrast, we expect that low doses (1/10 to 1/100 in comparison to oncology) of bromodomain inhibitor GNE-049 (or KAT inhibitor A-485) would be well-tolerated and have more favorable pharmacokinetics and toxicity profiles. While our data demonstrate the activity of compounds in primary dopaminergic neurons, mouse and human midbrain progenitors and dopaminergic neurons have distinct RNA expression profiles and species-specific dissimilarities.^33−35^ Many neuroprotective treatments successfully work in toxin-induced rodent models but not in human patients, failing at the stage of double-blinded randomized clinical trials,^36^ which substantiates the need for further studies in in vivo models and human neurons to scrutinize the potential of identified drug candidates.

Potential new therapies, technologies, repurposed drugs and small molecule compounds have been widely studied for PD,^37^ but currently available drug treatments of are only symptomatic. With the number of PD cases continuously increasing, the need for novel disease-modifying approaches in PD treatment is immense. Our findings suggest the potential for rational development of CBP/p300 inhibitors that modulate α-synuclein aggregation as a first-in-class therapeutic approach in PD via systemic administration. Thus, further repurposing studies of specific CBP/p300 inhibitors, which were initially developed for cancer therapy, for the treatment of Parkinson’s disease are warranted.

## Methods

### Primary Dopaminergic Neurons

To investigate the effect of the bromodomain inhibitors on the accumulation of the phosphorylated α-synuclein (at Ser129), Lewy pathology was modeled in primary neuronal culture with the application of exogenously prepared α-synuclein preformed fibrils. The workflow was based on our established α-synuclein aggregation assay in primary dopaminergic neurons.^24,25^

We treated primary midbrain cell culture, plated on poly-l-ornithine-coated 96-well plates, with mouse PFFs (StressMarq #SPR-324) on DIV-8. After 4 days of incubation with PFFs, we administered compounds or GDNF as a positive control and DMSO vehicle and dopaminergic medium as negative controls. On DIV-15, we fixed the cells and applied immunofluorescent staining for tyrosine hydroxylase and phosphorylated α-synuclein, to visualize and analyze Lewy-body-like intracellular accumulations in TH-positive dopamine neurons.

### Compounds

KAT and bromodomain inhibitors used in the present study were purchased from MedChemExpress (minimum purity >98%), including A-485 (CAS no. 1889279-16-6) and GNE-049 (CAS no. 1936421-41-8); Sigma-Aldrich (minimum purity ≥98%), including SGC-CBP30 (product no. SML1133), (+)-JQ1 (product no. SML1524), and PFI-3 (product no. SML0939); and Enamine Ltd. (minimum purity >95%), including 3i-5001 (product no. Z3159666854). Compounds were dissolved in 100% DMSO and then diluted in dopaminergic cell medium to the required concentrations prior to administration to cell cultures.

### Computational Chemistry

The commercial modeling package MOE 2019.0104 (Chemical Computing Group Inc., Montreal, Canada; http://www.chemcomp.com) was applied for protein preparation and molecular properties of the compounds, such as molecular weight, cLogP, and TPSA. An Amber12:EHT force field was applied for the molecule parametrization and protein structure preparation from Protein Data Bank (PDB).

### Data Analysis and Statistics

Plates were scanned with an automated microscope for well plates (Image Xpress Nano Automated Imaging System, Molecular Devices, LLC, San Jose, California), and acquired images were analyzed with the CellProfiler and the CellProfiler Analyst software packages.^25^ For statistical analysis of collected data, we used two-way repeated-measures ANOVA followed by Holm-Sidak’s multiple-comparisons test. All statistical analyses were carried out on unnormalized data in GraphPad Prism version 8.0.2 for Windows (GraphPad Software, San Diego, California USA, www.graphpad.com). The statistical significance threshold was set at *p* < 0.05 (corrected for multiple comparisons). The experimental data are presented as mean ± SD.

## Supporting Information Available

Results of A-485, PFI-3, 3i-5001 and (+)-JQ1 (Supplementary Figures 1 and 2), and combination of SGC-CBP30 and GDNF (Supplementary Figure 3) as well as A-485 and GNE-049 (Supplementary Figure 4) on the phosphorylated α-synuclein in dopaminergic neurons (PDF)
