# Supplementary material for: Domain-Independent Inhibition of CBP/p300 Attenuates α-Synuclein Aggregation
Source: ACS Chem Neurosci. 2021 Jun 10;12(13):2273–9. doi: 10.1021/acschemneuro.1c00215 (PMC8291498; doi:10.1021/acschemneuro.1c00215)
Supplement: Supplementary file 1 — cn1c00215_si_001.pdf [file cn1c00215_si_001.pdf]

Supplementary information

Domain-Independent Inhibition of CBP/p300 Attenuates Alpha-Synuclein Aggregation

Irena Hlushchuk[1], Heikki Ruskoaho[1], Andrii Domanskyi[2], Mikko Airavaara[1,3*], and Mika J. Välimäki[1*]

[1] *Drug Research Program, Division of Pharmacology and Pharmacotherapy, Faculty of Pharmacy, University of Helsinki, Finland*

[2] *Institute of Biotechnology, HiLIFE, University of Helsinki, Finland*

[3] *Neuroscience Center, HiLIFE, University of Helsinki, Finland*

[*] Corresponding authors:

Mikko Airavaara, Division of Pharmacology and Pharmacotherapy, Faculty of Pharmacy, Neuroscience Center, HiLIFE, University of Helsinki, P.O. Box 56, FI-00014 Helsinki, FINLAND
Email: mikko.airavaara@helsinki.fi

Mika J. Välimäki, Division of Pharmacology and Pharmacotherapy, Faculty of Pharmacy, University of Helsinki, P.O. Box 56, FI-00014 Helsinki, FINLAND
Email: mika.valimaki@helsinki.fi

Contents:

Supplementary Figures S1–S4, pages S2–S5

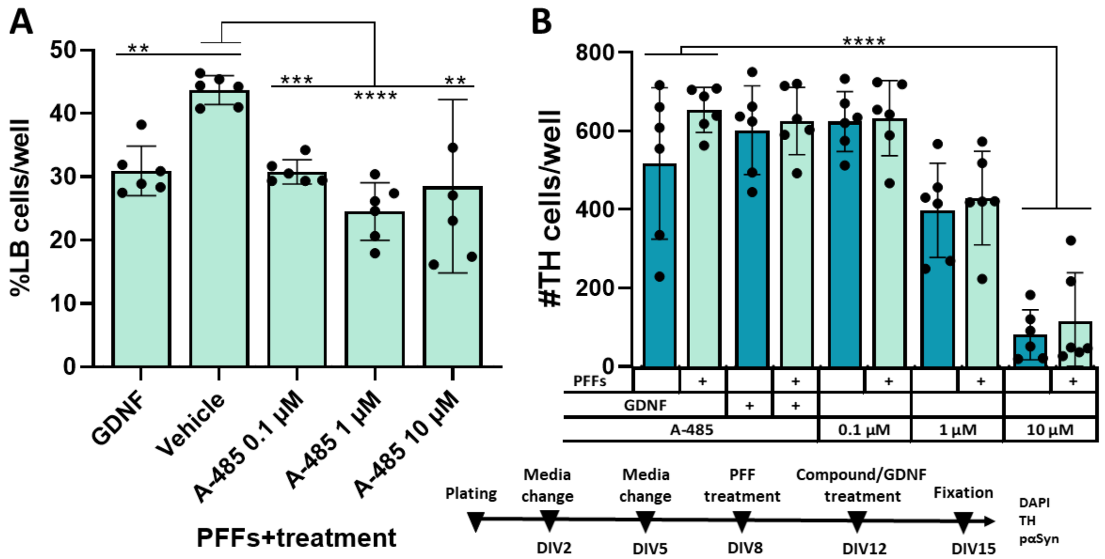

Supplementary Figure S1. *Compound A-485 reduced the intracellular phosphorylated α-synuclein accumulation and the survival of TH-positive neurons. (A) Percentage of TH-positive neurons, accommodating Lewy body-like accumulations. (B) Average number of TH-positive cells per well. (Two-way ANOVA, Tukey's multiple comparison test, ****p<0.0001, ***p<0.001, **p<0.01, n=6 wells of 2 independent experiments, data are mean ±SD).*

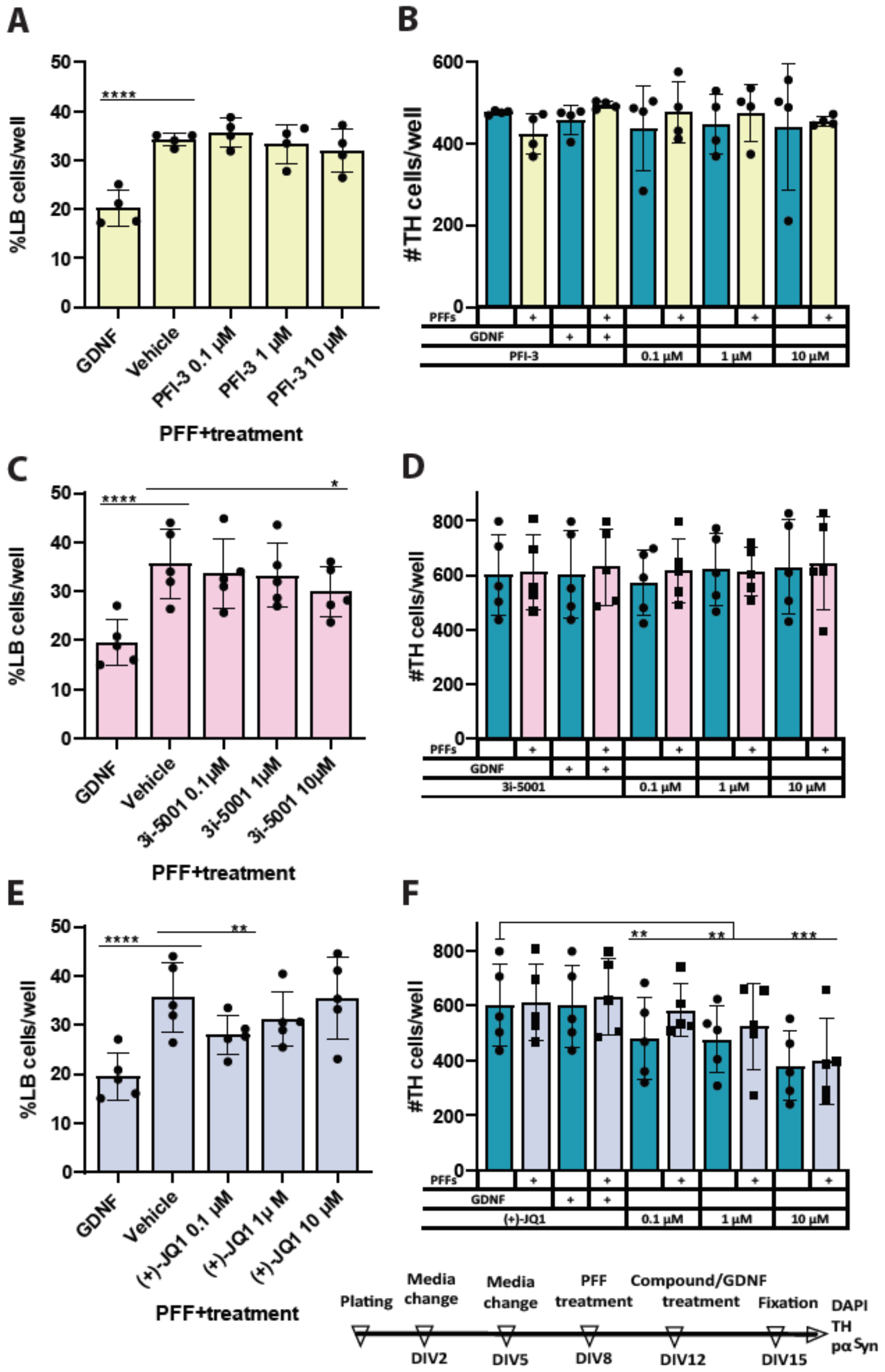

Supplementary Figure S2. *The effect of the bromodomain inhibitors for SMARCA2/4 (PFI-3), TAF1 (3i-5001) and BET ((+)-JQ1) on the level of intracellular phosphorylated α-synuclein accumulation in dopaminergic neurons. The toxicity of the presented concentrations to dopaminergic neurons is showed in comparison to the untreated cells in number of dopaminergic cells per well (B, D, E). The compounds at concentrations 0.1 µM, 1 µM, and 10 µM showed minor to no effect on the formation of aggregates in neurons (A, C, E). (Two-way RM ANOVA, Tukey's multiple comparison test, ****p<0.0001, ***p<0.001, **p<0.01, *p<0.05, n=4-5 independent experiments, data are mean ±SD).*

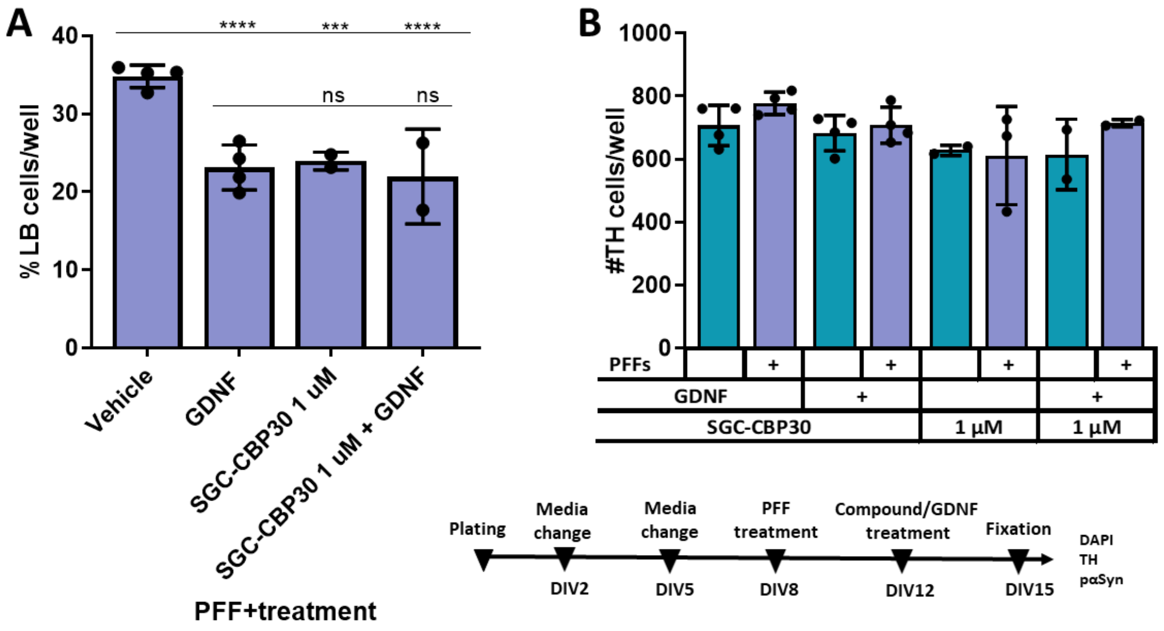

Supplementary Figure S3. *The combination of CBP/p300 inhibitor SGC-CBP30 and GDNF showed no synergistic decrease of intracellular phosphorylated α-synuclein accumulation in dopaminergic neurons. (A) The effect of the combination of SGC-CBP30 and GDNF on the formation of aggregates in dopaminergic neurons' soma is close to the effect of GDNF. (B) Selected concentrations are not significantly toxic to dopaminergic neurons. (Two-way ANOVA, Tukey's multiple comparison test, ****$p<0.0001$, ***$p<0.001$ n=2-4 technical repeats of 1 independent experiment, data are mean ±SD).*

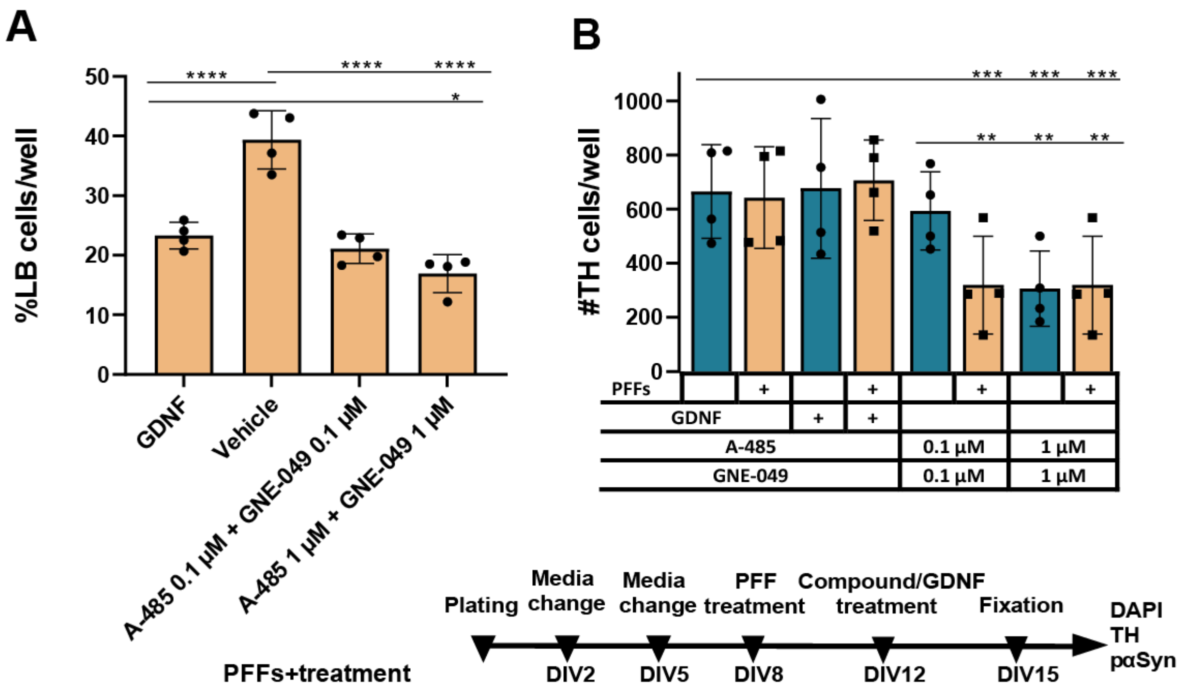

Supplementary Figure S4. *The combination of CBP/p300 inhibitors A-485 and GNE-049 showed decrease of phosphorylated α-synuclein accumulation and increase of cellular toxicity in dopaminergic neurons (A) The effect of the combination of A-485 and GNE-049 on the formation of α-synuclein aggregates in dopaminergic neurons. (B) Concentrations of A-485 and GNE-049 used were significantly toxic to dopaminergic neurons. (Two-way ANOVA, Tukey's multiple comparison test, ****p<0.0001, ***p<0.001, **p<0.01, *p<0.05, n=2-4 technical repeats of 4 independent experiments, data are mean ±SD).*
